# Liposome-Encapsulated *Escherichia coli* Lysates to Reconstitute Intracellular Macromolecular Crowding Effects

**DOI:** 10.1021/acssynbio.4c00824

**Published:** 2025-02-20

**Authors:** Milara S. Kalacheva, Nuno R. da Silva, Arnold J. Boersma

**Affiliations:** † Cellular Protein Chemistry, Bijvoet Centre for Biomolecular Research, Faculty of Science, Utrecht University, Utrecht 3584 CH, The Netherlands; ‡ DWI-Leibniz Institute for Interactive Materials, Aachen 52074, Germany; § CEB - Centre of Biological Engineering, Universidade do Minho, Campus de Gualtar, 4710-057 Braga, Portugal; ∥ LABBELS - Associate Laboratory in Biotechnology, Bioengineering, and Microelectromechanical Systems, Braga 4710-057, Portugal

**Keywords:** macromolecular crowding, giant unilamellar vesicles, lysate, cosolutes, FRET sensor, hyperosmotic stress

## Abstract

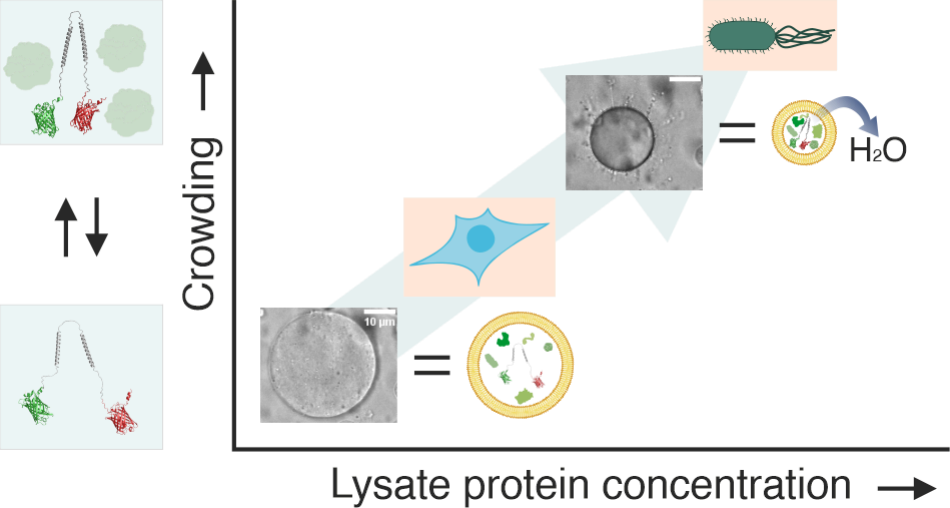

Intracellular macromolecular crowding impacts biomacromolecule behavior, including oligomerization, phase separation, and diffusion. However, understanding crowding effects in cells is challenging as cells respond and adapt to perturbations. Therefore, replicating in-cell crowding in liposomes would provide a good alternative to studying the consequences of macromolecular crowding. Here, we achieve physiological macromolecular crowding levels using *Escherichia coli* lysates in liposomes, as verified with a macromolecular crowding sensor. We shrink liposomes with a gradient-wise osmotic upshift to reach the high macromolecular crowding effects. We see that lysate induces higher macromolecular crowding than BSA at the same mg/mL, showing the need to use lysates to replicate in-cell behavior. We study the consequences of small cosolutes on macromolecular crowding and see that sugars and ATP modulate the lysate macromolecular crowding, implying they would also affect macromolecular crowding in cells. These artificial cells display the same crowding as *E. coli* at 220–300 mg/mL lysate and the same crowding as HEK293T at 50–100 mg/mL lysate. Hence, these artificial cells are a platform for obtaining information on physiologically relevant macromolecular crowding effects in a controlled environment.

## Introduction

Cells maintain high concentrations of macromolecules, where a range of 50–400 mg/mL is usually assumed to be physiological.^1−3^ These molecules take up space, and their steric repulsion excludes volume, which is called macromolecular crowding.^4^ Crowding excludes volume, which increases the effective concentration of a specific macromolecule. Thereby, association equilibriums shift to self-assembled or bound states, for example. Macromolecular crowding depends on the crowder size, number density, and shape. Macromolecular crowding also depends on the interaction between crowders or crowder organization, as this would, for example, reduce the crowder number density. The dependence on both crowder properties and organization means that in-cell crowding depends on an exceptionally large number of parameters that are challenging to untangle directly in cells,^5^ and understanding would benefit from controlled reconstitution in artificial cells.

Traditionally used crowding agents such as synthetic polymers (polyethylene glycol and Ficoll) or natural polymers and proteins (Dextran and bovine serum albumin) do not represent the intracellular situation well. Moreover, a high concentration of a single crowder leads to crowder-specific effects that are unlikely to be relevant for the cell. Closer-to-physiological crowding would be concentrating the actual components from living cells to reach *in vivo* macromolecular crowding. There are various instances where lysates have been concentrated to the same concentration as living cells (in mg/mL).^6^ Because concentrated lysates are highly viscous,^7^ concentrated lysates are more accessible by incorporating them in liposomes or similar compartments and shrinking them by osmotic pressure. Accordingly, increasing lysate concentrations dramatically increases *in vitro* transcription translation rates.^8,9^ However, concentrating lysates does not automatically imply that crowding behavior of living cells has been achieved^7,10^ because macromolecular crowding depends on the crowder organization, while the lysate physical properties will depend on the lysis and concentration method. Thus, while lysates can be concentrated, it has remained unclear if physiological macromolecular crowding can actually be achieved.

**Figure 1:**
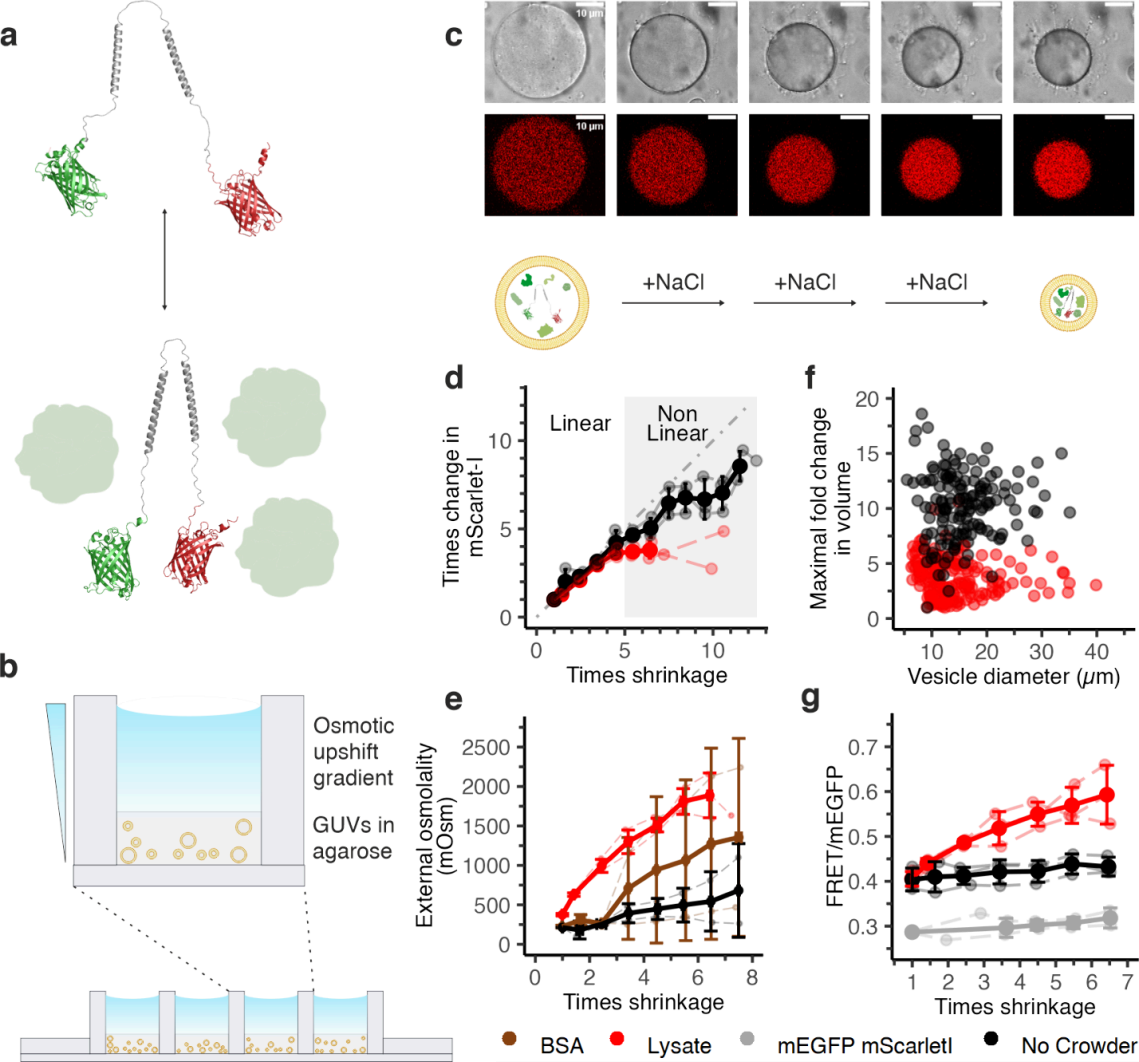
Response of single GUVs to increases in external osmolality. (a) The genetically encoded crGE2.3 (mEGFP/mScarlet-I) crowding sensor used to measure crowding in the vesicles. Crowding compresses the probe, thereby increasing FRET efficiency. (b) GUVs immobilized in agarose (0.5% w/v) in a chambered coverslip. The osmolality of the outer solution is slowly increased by adding NaCl solution in the top solution, which gradually diffused into the agarose gel. (c) Response of an immobilized GUV containing lysate and crGE2.3 to the osmotic upshift. (d) Fluorescence of mScarlet-I (FRET acceptor direct excitation and emission) correlates linearly (gray dotted line) with shrinkage up to 5 times shrinkage. (e) Shrinkage of a GUV depends on the external osmolarity, but not to the same extent for each crowder. (f) While GUVs containing lysate are smaller than ones without crowder, the amount of shrinkage is independent of the initial GUV size. Dots are transparent and darken when overlaid. (g) The FRET/donor emission ratio of crGE2.3 (red) increases in the presence of lysate but not in the absence of crowder (black). mEGFP and mScarlet-I, as two separate molecules without a linker (gray), do not show a FRET/donor increase. Error bars represent the standard deviation of the averages of 3 independent experiments. Independent experiments are connected with dashed lines.

We previously developed a method to measure macromolecular crowding, which was based on a genetically encoded Förster resonance energy transfer (FRET) sensor, crGE2.3 (Figure 1a).^11,12^ This sensor contains a linker with two α-helices linking a monomeric enhanced green fluorescent protein (mEGFP, donor) and mScarlet-I (acceptor). In uncrowded environments, the sensor remains in a relaxed conformation; in crowded environments, it is compressed, increasing the FRET efficiency. An advantage of a genetically encoded sensor is that it can be measured purified in buffer as well as measured when expressed in cells. Therefore, it provides a direct comparison between different crowded environments.

Here, we use crGE2.3 to monitor macromolecular crowding during the shrinkage of giant unilamellar vesicles (GUVs) filled with bacterial cell lysate. A reproducible and reliable crowding increase could be achieved through a gradient increase of the external osmotic pressure while monitoring the sensor at the single liposome level. We thereby gain insight into the crowding in living cells, the difference with purified protein crowders, and the influence of small molecules.

## Results and Discussion

### Model System to Replicate the Crowded Intracellular Environment

To recreate the crowded intracellular environment, we prepared bacterial cell lysate following a protocol adapted from Fujiwara et al.^6^ (see Materials and Methods). The lysate concentrations ranged from 60 to 80 mg/mL protein in a lysis buffer (30 mM potassium glutamate, 6 mM magnesium glutamate, 10 mM sodium phosphate buffer (NaPi, pH 7.4), and a cOmpleteTM protease inhibitor cocktail). We encapsulated freshly prepared lysate in GUVs to replicate the crowded intracellular confinement. The GUVs were created using the emulsion transfer method,^13^ providing a range of vesicle sizes. Each GUV contained the crowding sensor, 50 mg/mL lysate, and 0.1 M sucrose to stabilize the vesicles. The external glucose solution in 10 mM NaPi, pH 7.4 was isosmotic. The vesicles were immobilized in 0.5% w/w low melting agarose for observation at the single-vesicle level (Figure 1b,c).^14^ Single-vesicle observation provided the relative shrinkage for each vesicle. Thereby, we determined the final lysate concentration despite the distribution in vesicle sizes.

To increase lysate concentration from 50 mg/mL to physiological levels, we gradually increased the osmolarity by titrating NaCl on top of the gel with the immobilized GUVs. The salt was then allowed to diffuse into the gel (Figure 1b,c). This salt gradient prevented content leakage from the vesicles and maintained the lysate soluble. Indeed, sudden stepwise hyperosmotic upshifts led to phase separation of the lysate visible by microscopy (Figure S1b), and a lack of the FRET increase.

Next, we verified the membrane stability by plotting the increase in mScarlet-I fluorescence versus the decrease in vesicle volume (Figures 1d and S1c). Both lysate-crowded and noncrowded vesicles maintained linearity up to a 5-fold decrease in volume. Beyond this point, fluorescence emission was lower than expected. The linearity upon concentrating from 50 to 250 mg/mL indicates reliable sensor readouts within the expected physiological crowding range.

Because crowders should increase osmolality via colloidal osmotic pressure, we compared the external osmolality required to shrink the vesicles (Figure 1e). Vesicles containing crowders required higher external osmolality to achieve the same shrinkage as vesicles without crowders. Hence, the macromolecules affect the vesicle’s response to osmotic stress. In addition, vesicles containing cell lysates require a higher external osmolarity to shrink than those containing BSA. Possibly, BSA self-association lowers its colloidal osmotic pressure, making it easier to shrink the vesicles. This aligns with our previous observation where shrinkage of Ficoll-loaded w/o/w emulsions required higher osmolarity than emulsions containing BSA.^15^ Importantly, the relative vesicle shrinkage was independent of its initial size within an experiment (Figure 1f).

We monitored the crowding sensor by laser scanning confocal microscopy. The FRET emission (ex. 488 nm, em. 600–700 nm) divided by the donor emission (ex. 488 nm, em. 510–525 nm) increased with vesicle shrinkage in lysate-loaded vesicles from 0.41 ± 0.02 to 0.60 ± 0.07 (Figures 1g and S1a). In contrast, the ratio in buffer-only vesicles showed a marginal increase from 0.40 ± 0.03 to 0.45 ± 0.02, likely due to intermolecular FRET, as we noted before.^15^ To test if the increase in ratio in the presence of lysate was due to FRET, we compared our data with unlinked mEGFP and mScarlet-I that should not FRET. Indeed, the ratio was lower, with a small increase from 0.30 ± 0.01 to 0.32 ± 0.02 upon shrinkage, likely due to intermolecular FRET as proposed above. Hence, the increase in intramolecular FRET for crGE2.3 follows the proposed compression due to macromolecular crowding. Thus, we created stable GUVs with lysate-induced macromolecular crowding that can be increased by applying a salt gradient.

### Crowding Depends on the Crowder and Its Solubility

**Figure 2:**
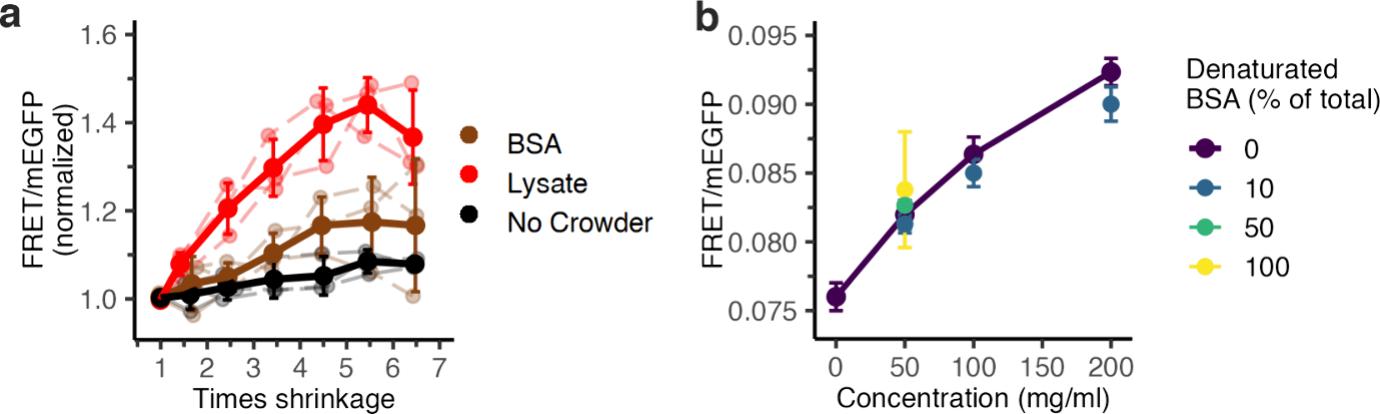
Macromolecular crowding depends on the crowding agent. (a) FRET/mEGFP (normalized to isosmotic conditions) of lysate-crowded (red), BSA-crowded (brown), or uncrowded GUVs (black) plotted against the degree of shrinkage of these GUVs showing crowder-dependence. (b) FRET/mEGFP dependence on BSA concentration. BSA solutions containing 0%, 10%, 50%, or 100% heat-denatured BSA. Error bars represent the standard deviation of the averages of 3 independent experiments. Independent experiments are connected with dashed lines.

BSA is a model crowder because it is a globular protein and readily available in large quantities. Since we observed a different propensity of vesicles to shrink when loaded with BSA, we compared BSA-induced crowding with lysate-induced crowding. We encapsulated 50 mg/mL BSA within our GUVs and measured the crowding with the crGE2.3 sensor. We saw that at the same protein weight%, the FRET efficiency was lower in BSA-crowded GUVs compared to lysate-crowded GUVs, indicating lower crowding (Figure 2a). Specifically, the increase in the FRET/mEGFP ratio was 1.17 ± 0.07 for BSA-crowded GUVs and 1.4 ± 0.1 for lysate-crowded GUVs at 4.5-fold shrinkage. A possible explanation is that BSA-induced macromolecular crowding is lower due to its tendency to self-associate, similar to our reasoning why these vesicles shrink more (see above). While lysates will also contain a significant mass of rRNA, their number density will be ∼100-fold lower,^16^ and we expect direct crowding effects to originate primarily from the lysate proteins. Therefore, BSA is a less efficient crowder than cell lysates.

The crowding of cell lysates is potentially affected by the folding of its constituent proteins. Unfolded proteins take up more volume than folded proteins and may increase crowding unless they aggregate. Therefore, we aimed to determine the role of crowder denaturation on macromolecular crowding. To this end, we mixed native BSA with heat-denatured BSA in bulk in the presence of crGE2.3. BSA denaturation was confirmed with DLS measurements (Figure S5b). Increasing native BSA from 0 to 200 mg/mL increases the FRET ratio from 0.076 ± 0.001 to 0.092 ± 0.001 (Figure 2b), while exchanging 10% of the native BSA with denatured BSA provided slightly lower ratios. Hence, unfolding BSA somewhat lowers the crowding. The solution turbidity also increased (Figure S5a), suggesting aggregation (or precipitation), which could lower the macromolecular crowding. We conclude that protein denaturation does not increase crowding per se, likely due to increased aggregation.

### Small Cosolutes Modulate Macromolecular Crowding

**Figure 3:**
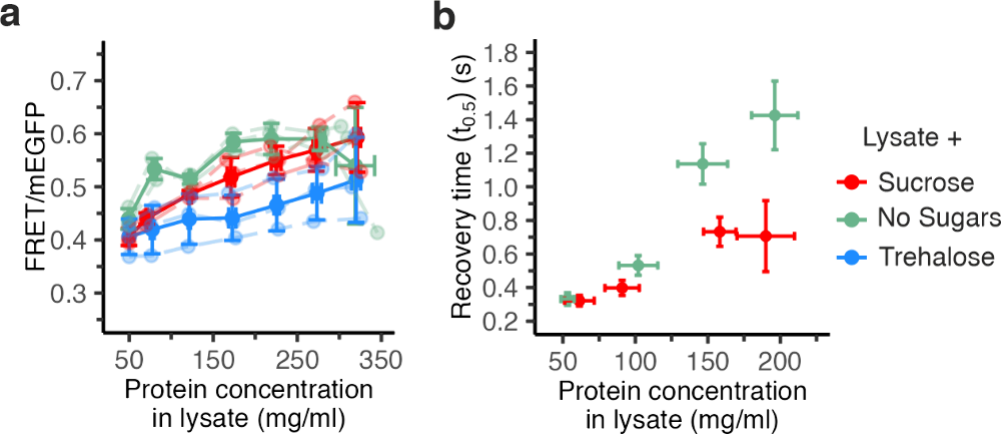
Sugars modulate macromolecular crowding induced by lysates. (a) Lysate-crowded GUVs containing either sucrose (starting concentration 0.1 M) (red) or trehalose (starting concentration 0.1 M) (blue) have lower FRETs/mEGFP than vesicles containing no sugars (green) at the same total protein lysate concentration; (b) FRAP experiment follows the measured crowding, showing the average time needed for lysate-crowded GUVs containing sucrose is shorter than in the absence of sugars. Error bars represent the standard deviation of the averages of 3 independent repeats. Independent experiments are connected with dashed lines.

Next, we investigated the impact of sugars on our lysate-crowded system. We initially incorporated sucrose to improve membrane stability.^17,18^ However, sugars could affect macromolecular crowding by (i) changing crowder conformation or hydration state or (ii) changing macromolecular crowder number density by modulating crowder–crowder interactions: sucrose and trehalose have been described to stabilize protein folding and inducing protein self-assembly.^19^ Thus, we added 0.1 M sucrose or trehalose to 50 mg/mL lysate-containing GUVs and compared this to the absence of added sugars. These GUVs were osmotically shrunk as before. We saw that GUVs without sugars had the highest ratios, followed by sucrose and trehalose (Figure 3a). At around 70 mg/mL (around 1.4-fold shrinkage), the FRET/mEGFP ratios were 0.53 ± 0.02 in the absence of sugars, 0.44 ± 0.01 for sucrose, and 0.42 ± 0.05 for trehalose. The differences became more pronounced at 170 mg/mL lysate (3.4-fold shrinkage) with 0.58 ± 0.02 in the absence of sugars, 0.52 ± 0.04 for sucrose, and 0.44 ± 0.04 for trehalose. At 5-fold shrinkage, 250 mg/mL, the differences were less as the crowding increase leveled off in the absence of sucrose. Hence, sugars reduce macromolecular crowding.

We hypothesized that if sugars reduce crowding through increasing crowder–crowder interactions, the diffusion of a test particle would increase with sugars. This contrasts with the increased viscosity when dissolving sugars at high concentrations. We thus measured the sensor diffusivity by fluorescence recovery after photobleaching (FRAP). We saw that the lysate-crowded sensor in vesicles containing sucrose indeed displayed faster recovery times (*t*_0.5_ = 0.7 ± 0.3 s at 158 ± 11 mg/mL) than those without sucrose (*t*_0.5_ = 1.4 ± 0.5 s at 147 ± 17 mg/mL) (Figure 3b), implying higher diffusivity. Hence, the lower crowding with sucrose aligns with higher diffusivity despite having the same mg/mL lysate, from which we infer that sucrose likely alters crowder organization.

To exclude that sucrose reduces crowding by inducing lysate aggregation (or precipitation), we assessed the turbidity of dense lysates in bulk with and without sucrose using Pur-A-Lyzer Midi Dialysis tubes. We find that the turbidities are similar: sucrose induced slightly lower turbidity (277 ± 17 mAu at 91 ± 6 mg/mL) compared to lysates without sucrose (310 ± 16 mAu at 87 ± 6 mg/mL) (Figure S4a). Therefore, lysate aggregation does not explain the difference in crowding effects. To determine if sucrose affected the sensor directly, we titrated sucrose to crGE2.3 in buffer. The FRET ratios display a much smaller decrease than in the liposomes upon adding 0.4 M sucrose (Figure S3), and we can exclude that direct sucrose interaction with the sensor plays a significant role. Hence, sucrose modulates macromolecular crowding induced by cell lysates, likely by changing its organization.

**Figure 4:**
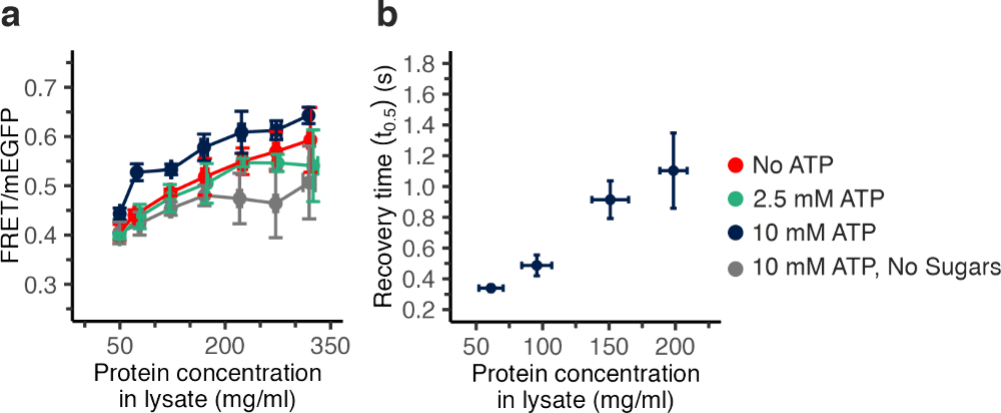
ATP modulates the macromolecular crowding of concentrated lysates. (a) FRET/mEGFP was plotted versus the total protein concentration in lysate-crowded GUVs containing starting concentrations of sucrose (0.1 M) and ATP (2.5 mM or 10 mM, green and dark blue respectively), ATP (10 mM) without sucrose (gray), or sucrose (0.1 M) without ATP (red); (b) FRAP measurement of lysate crowded GUVs containing ATP (10 mM) and sucrose (0.1 M). Error bars represent the standard deviation of 3 biological repeats.

Next, we tested adenosine triphosphate (ATP) as it was suggested to alter protein stability and disperse protein assemblies,^20,21^ which could thus change crowding as well. We started at 2.5 mM ATP in lysates to reach physiological concentrations of ∼10 mM ATP^22^ upon vesicle shrinking. We saw that ATP does not affect the FRET ratios significantly (0.55 ± 0.02 at 275 ± 3 mg/mL lysate; Figure 4a). When we instead started with 10 mM ATP, we saw a higher crowding indeed (0.61 ± 0.02 at 273 ± 7 mg/mL lysate). These ATP and sucrose effects do not compensate: ATP in the absence of sucrose gives a lower ratio (0.46 ± 0.07 at 272 ± 3 mg/mL) (Figure 4a), implying a complex mechanism.

To obtain insight into the effect of ATP on macromolecular crowding, we measured FRAP. We saw that recovery with 10 mM ATP is slower than without ATP and faster than the recovery without ATP and sucrose (*t*_0.5_ = 1.1 ± 0.3 s at 198 ± 10 mg/mL) (Figures 4b and 3b). This observation aligns with our hypothesis that when crowding increases, recovery is slower. Here, we assume that lysate effects dominate over viscosity changes from ATP itself, similarly as we noted for sucrose. The turbidity of lysates concentrated in the presence of 10 mM ATP and 0.1 M sucrose remained rather similar compared to the absence of ATP, suggesting no extensive crowder solubility change required to change crowding (Figure S4b and denatured BSA experiments above). Moreover, ATP does not affect the sensor directly (Figure S3b). Hence, incorporating high ATP concentrations beyond physiological concentrations, in combination with sucrose, enhances macromolecular crowding.

We tested the effect of a few other additives. The small zwitterionic protein-protective osmolytes betaine and trimethylamine N-oxide (TMAO) did not significantly influence macromolecular crowding (Figure S2c,d). The FRET ratio is somewhat reduced when using 40 mM instead of 6 mM magnesium glutamate (Figure S2b). When we used lysates from cells adapted to grow with a 300 mM NaCl osmotic upshift, we observed the same crowding as lysates from cells grown in a regular LB medium (Figure S2a). In conclusion, small molecules can change macromolecular crowding, and in the case of sucrose and ATP, this readout corresponds to a concomitant change in probe diffusion.

### Direct Macromolecular Crowding Comparison between Artificial Cells and Living Cells

**Figure 5:**
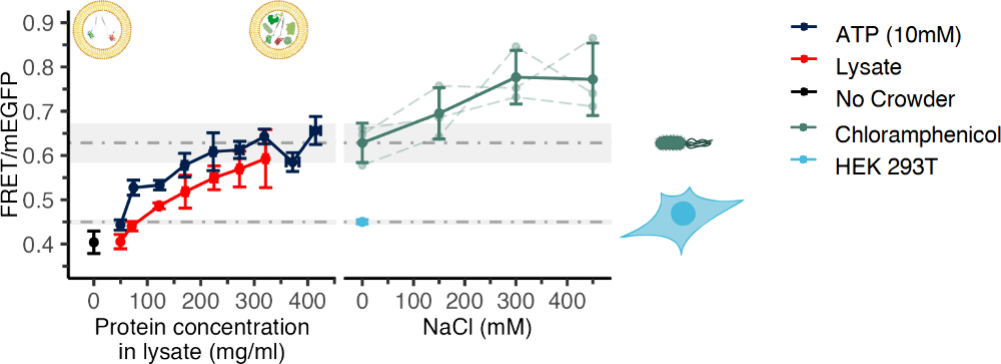
Comparison of lysate-crowded GUVs with the crowding of cells. Left: FRET/mEGFP of osmotically shrunken GUVs containing no crowder (black), lysate (red), or lysate with 10 mM ATP starting concentration (dark blue), plotted against the total protein concentration in the GUVs. Data is from Figure 4a. Right: FRET/mEGFP of osmotically stressed *E. coli* cells (green) treated with 0.2 mg/mL chloramphenicol, and HEK293T cells (light blue dot). Dotted lines and gray shading represent the average ratio and standard deviation for unstressed cells. Error bars represent the standard deviation of 3 biological repeats. Independent experiments are connected with dashed lines.

Next, we tested if macromolecular crowding in the artificial cells compares to living cells to (i) indicate if we achieved physiological crowding levels and (ii) assess how crowded living cells are, based on their protein content. To measure the FRET ratios in living cells, we expressed crGE2.3 in *E. coli* BL21(DE3).^11^ In contrast to *in vitro* experiments, the maturation of the fluorescent proteins in *E. coli* is incomplete.^23^ Therefore, we treated the cells with chloramphenicol to stop translation, allowing the fluorescent proteins to mature and directly compare the ratios with purified protein in *in vitro* experiments. We used the same microscope settings as the artificial cells for direct ratio comparison. Chloramphenicol treatment increased the FRET ratios as the acceptor matured maximally (Figure S6). Applying an osmotic upshift by adding NaCl to the medium showed the expected FRET ratio increase from 0.62 ± 0.04 to 0.77 ± 0.08 upon osmotic upshift (Figure 5, right green line). We previously noted that adding sucrose instead of NaCl provided similar results,^11,24^ excluding NaCl-specific effects.

Next, we compared the in-cell macromolecular crowding with that of artificial cells. Besides using the same microscopy settings and maximal fluorescent protein maturation, the sensor’s insensitivity to relevant cellular molecules facilitates the comparison.^11,24^ In addition, lysates have a similar composition as the cytosol. We saw that artificial cells had reached the same ratios as *E. coli* within the biological variation of the experiments. This indicates that the artificial cells reach physiologically relevant macromolecular crowding. The macromolecular crowding in artificial cells with a high ATP concentration corresponds to 225–300 mg/mL cell lysate based on protein content, which compares with literature values for *E. coli* (∼280–300 mg/mL).^25^ Lysates without ATP reach the window of in-cell crowding at ∼300 mg/mL lysate protein. We cannot increase the lysate crowding further, and this aligns with our observations that the fluorescence increase of mScarlet-I is not linear with the decrease in cell volume after 250–300 mg/mL lysate (Figure 1f). This has been seen before for GFP fluorescence in lysates,^6^ and correlates with a dramatic increase in lysate viscosity,^7^ which could be due to an altered physical state of the lysates. Apparently, additional lysate or membrane components are needed for higher crowding levels. Nonetheless, concentrating cell lysates provide macromolecular crowding levels of *E. coli* and a similar protein concentration in mg/mL.

Since our artificial cells provide relevant macromolecular crowding levels, we assessed how crowding in mammalian cells compared to *E. coli*. The macromolecular crowding in mammalian cells is reported to be lower than in bacterial cells.^1^ We measured the crowding in HEK293T cells stably expressing the crGE2.3 crowding sensor to our model system. Since HEK293T cells are dividing much slower than *E. coli*, we assumed the fluorescent proteins have fully matured. These cells exhibited a FRET ratio of 0.45 ± 0.01. It was previously shown that lysates from *E. coli* and mammalian cell lines have similar physical properties,^7^ and we thus compared HEK293T FRET ratios with our bacteria lysate-crowded GUVs and found that HEK293T crowding corresponded to 50–75 mg/mL in GUVs with 10 mM ATP, or 70–122 mg/mL in GUVs without ATP (Figure 5). We thus confirm with crGE2.3 that crowding is lower in a human cell line and that it is straightforward to reach HEK293T macromolecular crowding with *E. coli* lysates.

## Conclusion

In this study, we encapsulated *E. coli* lysates in liposomes to achieve the original in-cell macromolecular crowding levels. Our approach demonstrated that concentrated bacterial lysate can mimic the intracellular environment, providing a more accurate representation than traditional protein or polymer crowders. This system bridges the gap between *in vitro* and *in vivo* conditions by providing a complex heterogeneous cell-like environment that includes diverse surface chemistries critical to cellular function. The FRET-based crowding sensor, crGE2.3, enabled us to quantitatively compare crowding levels. The sensor showed that *E. coli* lysates reached crowding concentrations equivalent to those in *E. coli* and HEK293T cells. The observation that small molecules such as sucrose and ATP modulate crowding in artificial cells implies that they have consequences for macromolecular crowding in living cells: this would be challenging to investigate in living cells because changing the concentration of a molecule in a cell leads to a physiological response. Thus, these artificial cells with physiological crowding offer more control than intracellular studies, making them an excellent substitute for the native cytosol.

## Materials and Methods

### Gene Expression and Protein Purification

Chemically competent *E. coli* BL21(DE3) cells were transformed with plasmid pRSET A containing the crowding sensor crGE2.3 gene.^26^ The cells were grown in 200 mL Luria–Bertani (LB) medium (NaCl 10 g/L, tryptone 10 g/L, yeast extract 5 g/L) containing 50 μg/mL carbenicillin at 37 °C until the OD_600_ reached 0.6. Protein expression was induced overnight with 1 mM isopropyl β-D-1-thiogalactopyranoside (IPTG) at 28 °C and 180 rpm. The cells were harvested by centrifugation at 4000 g for 40 min and washed once with isosmotic sodium phosphate buffer (NaPi, pH 7.4). The cell pellet was then resuspended in lysis buffer (500 mM NaCl, 50 mM NaPi, pH 7.4, 6 mM MgCl_2_, 1 mg/mL lysozyme, cOmplete EDTA-free protease inhibitor cocktail), incubated at 4 °C for 30 min, and lysed with a high-pressure homogenizer (Constant Systems Ltd.) at 20 kpsi. The cell extract was treated with deoxyribonuclease I (DNase I) for 30 min, cleared by centrifugation at 4 °C for 30 min at 16000 g, and supplemented with 10 mM imidazole. The recombinant protein was purified using the NGC Discover 10 Chromatography System (BioRad) with a HisTrap FF column (Cytiva). The wash and elution buffers contained imidazole (20/250 mM), NaPi, pH 7.4 (50 mM), and NaCl (500 mM). The purified protein was analyzed using 10% SDS-PAGE. The fractions that showed pure protein were combined, and the buffer was exchanged to 10 mM NaPi, pH 7.4, aliquoted, and stored at −80 °C.

### Generation of Cell Lysates

A starter culture of *E. coli* BL21(DE3) was grown at 37 °C in 50 mL LB without a selection marker. Then, the culture was diluted in 1 l fresh LB media to OD_600_ 0.05 and grown at 37 °C and 180 rpm until OD600 of 0.9–1.0. The bacteria were harvested by centrifugation at 4000 g, washed once with isosmotic NaPi, pH 7.4, and the pellet was resuspended with lysis buffer. The cells were lysed by running the cell suspension twice through a high-pressure homogenizer (Constant Systems Ltd.) at 20 kpsi. DNase I was added, and the lysate was incubated for 30 min at room temperature. The lysate was centrifuged for 90 min at 21300 g at 4 °C. The final protein concentration of the soluble fraction was measured with the Pierce BCA Protein Assay. The lysates were used immediately or aliquoted and stored at −80 °C.

Depending on the experiment, the lysis buffer contained a combination of the following components: 10 mM NaPi, pH 7.4, 6 mM or 40 mM magnesium glutamate, 30 mM potassium glutamate, complete EDTA-free protease inhibitor cocktail, 0.1 or 0.25 M TMAO, 0.1 or 0.25 M betaine, 2.5 mM or 10 mM ATP.

### Generation and Immobilization of GUVs

Giant unilamellar vesicles (GUVs) were generated with the inverted emulsion method, also known as the water-in-oil emulsion-transfer method.^13,27^ In short, the oil phase consists of a mixture of 5 mg/mL 1-palmitoyl-2-oleoyl-*sn*-glycero-3-phosphocholine (POPC):1-palmitoyl-2-oleoyl-*sn*-glycero-3-phospho-(1′-rac-glycerol) (POPG):cholesterol (8.5:1:0.5 weight ratio) in liquid paraffin oil (FUJIFILM Wako). Depending on the experiment, the inner solution contained a combination of crowder (cell lysate, BSA, or none), lysis buffer, crGE2.3, and sugars (0.1 M sucrose, 0.1 M trehalose, or none). The crGE2.3 concentration should be at least 4 times the background fluorescence in all microscopy channels (see below). We have seen no dependence of the readouts in the sensor concentration range of ∼25–200 μg/mL. The outer solution was an isosmotic solution of glucose in 10 mM NaPi, pH 7.4. Osmolalities were measured with Gonotec Osmomat 3000.

An emulsion of inner solution and oil was created by mixing 30 μL inner solution in 300 μL oil phase. The emulsion was incubated for 10 min on ice, carefully layered on top of the outer solution, and incubated for an additional 10 min. Then, the GUVs were created by centrifugation for 5 min at 5000 g and 4 °C. The clear oil phase and most of the outer solution were carefully pipetted out so as not to disturb the pellet. A fresh outer solution was added, and the pellet was carefully resuspended. The generated GUVs were immobilized in agarose, as described in.^14^ Top Vision Low Melting Point Agarose (Thermo Scientific) was dissolved in NaPi, pH 7.4 to 4% (w/v). The molten agarose was mixed with the GUVs solution to create a 0.5% (w/v) final concentration of agarose. Then 200 μL of the GUV-agarose solution was placed in a chambered coverslip with 8 wells (Ibidi) and left to polymerize. Once the agarose was fully polymerized, a 200 μL outer solution was layered on top. Single GUVs were measured and then osmotically stressed by carefully adding 200 μL hyperosmotic NaCl solution on top. After 10 min of incubation, new pictures were taken of the same GUVs. The osmotic stress step was repeated until the GUVs showed significant shrinkage. The volume of the GUVs at each stage was determined by their diameter.

### Fluorescent Microscopy and FRET Measurements

Scanning confocal fluorescent microscopy was performed on Leica TCS SP8 with 63× (1.2 NA) water immersion objective and 3.5 Airy. The monomeric enhanced green fluorescent protein (mEGFP) was excited at 488 nm and detected at 510–525 nm emission (donor channel), whereas mScarlet-I was excited at 561 nm and detected within the 600–700 nm band (acceptor channel). The FRET channel was detected by excitation at 488 nm and detection in the 600–700 nm emission band. The detection of all channels was done with photomultiplier tube (PMT) detectors with 1000 gain. The background signal of the cell lysate was subtracted as indicated by GUV/cells without crGE2.3. Image quantification was done with either Leica Application Suite or with ImageJ Fiji.

### FRAP Measurements

FRAP experiments were conducted using a confocal laser scanning microscope, Carl Zeiss LSM880 Fast AiryScan, with a 63× glycerol immersion objective (1.2 NA). Images were captured at 128 × 128 pixel resolution, with a time series of 40 prebleach frames, 3 bleach cycles, and 100 recovery frames, totaling 340 frames at 51.61 ms per frame. A spherical region of interest (ROI) was bleached using a 561 nm laser at 100% power for 15 iterations. Recovery images were taken at 1% power, and detection was via a PMT detector.

For each condition, three vesicles were used for acquisition photobleaching measurement and three for lysate background measurement. At least seven vesicles per condition were photobleached. The lysate background was subtracted, and acquisition photobleaching was corrected. Frames 30–40 of the prebleach phase were used for normalization. Recovery curves were fitted using GraphPad Prism with a one-phase exponential association equation.

### Concentrating Cell Lysates in Bulk

Bacterial cell lysates were prepared as described above. These lysates were then concentrated via evaporation in Pur-A-Lyzer Midi Dialysis tubes. In short, 800 μL of lysate at a concentration of 50 mg/mL was placed in each dialysis tube and incubated at 4 °C in a ventilated room. At each time point (0, 18, 24, 30, and 43 h), aliquots were taken to measure protein concentration (Pierce BCA protein assay), refractive index, and turbidity.

### Preparation of Denatured BSA

BSA was dissolved in 10 mM NaPi, pH 7.4, and incubated overnight at 4 °C to create a stock BSA solution with a concentration of 300 mg/mL. Subsequently, further dilutions of the BSA stock with 10 mM NaPi, pH 7.4 were made to achieve final concentrations of 0, 50, 100, 150, and 200 mg/mL BSA. A 50 mg/mL BSA dilution was denatured by incubating it at 95 °C for 1 h at 1000 rpm, followed by cooling to room temperature. The denaturation was confirmed by dynamic light scattering (DLS) (Figure S5b). By mixing the denatured 50 mg/mL BSA solution with the nondenatured 300 mg/mL stock BSA, we prepared the BSA solutions used in Figure 2b. These solutions had final concentrations of 50, 100, 150, and 200 mg/mL BSA, with 10% of the BSA being denatured. For the solution with a final concentration of 50 mg/mL, additional solutions were prepared in which 50% or 100% of the BSA was denatured.

### Spectrofluorometer Measurements

The fluorescence spectrum of crGE2.3 was measured with an excitation wavelength of 465 nm and an emission range of 490–700 nm using a Varian Cary Eclipse Spectrophotometer and Quartz Glass High Performance cuvette (1 mL). First, the spectrum of the solution without crGE2.3 was measured to determine the background signal. Subsequently, crGE2.3 (25 μg/mL) was added, and the spectrum was measured again. The FRET/donor was calculated after subtracting the background and dividing the average emission in the 583–597 nm band (FRET emission) by the emission in the 505–515 nm band (donor emission). Additionally, the acceptor was directly excited at 569 nm, with the emission detected in the 580–700 nm band (acceptor emission).

### Turbidity and DLS During Thermal Unfolding

Turbidity and DLS measurements were performed using the Prometheus Panta (NanoTemper) with Prometheus high-sensitivity capillaries. Turbidity measurements were conducted at 50% laser power across a temperature range from 15 to 95 °C (unfolding stage) and from 95 to 15 °C (refolding stage). For the DLS measurements the samples were diluted to 2 mg/mL and measured at 20 °C and 50% laser power.

### *E. coli* Fluorescence Confocal Microscopy

*E. coli* BL21(DE3) was transformed with crGE2.3, plated on LB agar plates with carbenicillin (50 μg/mL), and then incubated overnight at 37 °C. Nontransformed *E. coli* BL21(DE3) cells were also plated on LB agar without a selection marker to control for autofluorescence. Single clones from both plates were grown in MOPS minimal medium^28^ overnight at 37 °C with shaking at 200 rpm. The overnight cultures were diluted in fresh medium to an OD_600_ of 0.015–0.025 and grown at 37 °C, 200 rpm, until an OD_600_ of 0.14–0.15. Cells were then transferred to two new flasks and split into control and chloramphenicol-treated groups (0.2 mg/mL), with nontransformed cells added to both. Both groups were incubated at 37 °C, 200 rpm, for 1.5 h. After incubation, aliquots from both groups were collected and washed twice in fresh MOPS medium (without potassium and glucose) by centrifugation at 3000 g for 1 min at 37 °C. NaCl was added to induce osmotic stress to cells in the absence of potassium, and the cells were placed on coverslips. Images were taken as described above within 5 min of NaCl addition.

### Mammalian Cell Culture

Human embryonic kidney 293T (HEK293T) cells and HEK293T cells expressing crGE2.3 were cultured in Dulbecco’s Modified Eagle Medium (DMEM) supplemented with 10% fetal bovine serum (FBS) and 1% penicillin-streptomycin at 37 °C with 5% CO_2_. For microscopy analysis, the cells were mixed and plated at a density of 190,000 cells per well in an 8-well chambered coverslip (Ibidi). The cells were incubated overnight at 37 °C with 5% CO_2_. The next day, the old medium was replaced with fresh media (DMEM + 10% FBS + 1% penicillin–streptomycin, no phenol red). Fluorescent images were obtained as described above, with the cells maintained at 37 °C during imaging.

## Supporting Information Available

Characterization of liposome shrinkage and FRET, effects of cosolutes on macromolecular crowding, direct interaction of cosolutes with the FRET sensor, turbidity measurements of lysates, unfolding and DLS of BSA, the role of chloramphenicol on crowding in *E. coli*, and the list of DNA sequences of the genes encoding the crGE2.3 for *E. coli* and HEK293T (PDF)
