# Supplementary material for: Liposome-Encapsulated Escherichia coli Lysates to Reconstitute Intracellular Macromolecular Crowding Effects
Source: ACS Synth Biol. 2025 Feb 20;14(3):901–8. doi: 10.1021/acssynbio.4c00824 (PMC11934223; doi:10.1021/acssynbio.4c00824)
Supplement: Supplementary file 1 — sb4c00824_si_001.pdf [file sb4c00824_si_001.pdf]

Supplementary Information

Liposome-Encapsulated *Escherichia coli* Lysates to Reconstitute Intracellular Macromolecular Crowding Effects.

Milara S. Kalacheva,[1,2] Nuno R. da Silva,[1,3,4] and Arnold J. Boersma[1,2]*

[1] Cellular Protein Chemistry, Bijvoet Centre for Biomolecular Research, Faculty of Science, Utrecht University, Utrecht 3584 CH, the Netherlands.

[2] DWI-Leibniz Institute for Interactive Materials, Aachen 52074, Germany

[3] CEB - Centre of Biological Engineering, Universidade do Minho, Campus de Gualtar, 4710-057 Braga, Portugal

[4] LABBELS - Associate Laboratory in Biotechnology, Bioengineering, and Microelectromechanical Systems, 4710-057 Braga, Portugal

*a.j.boersma@uu.nl

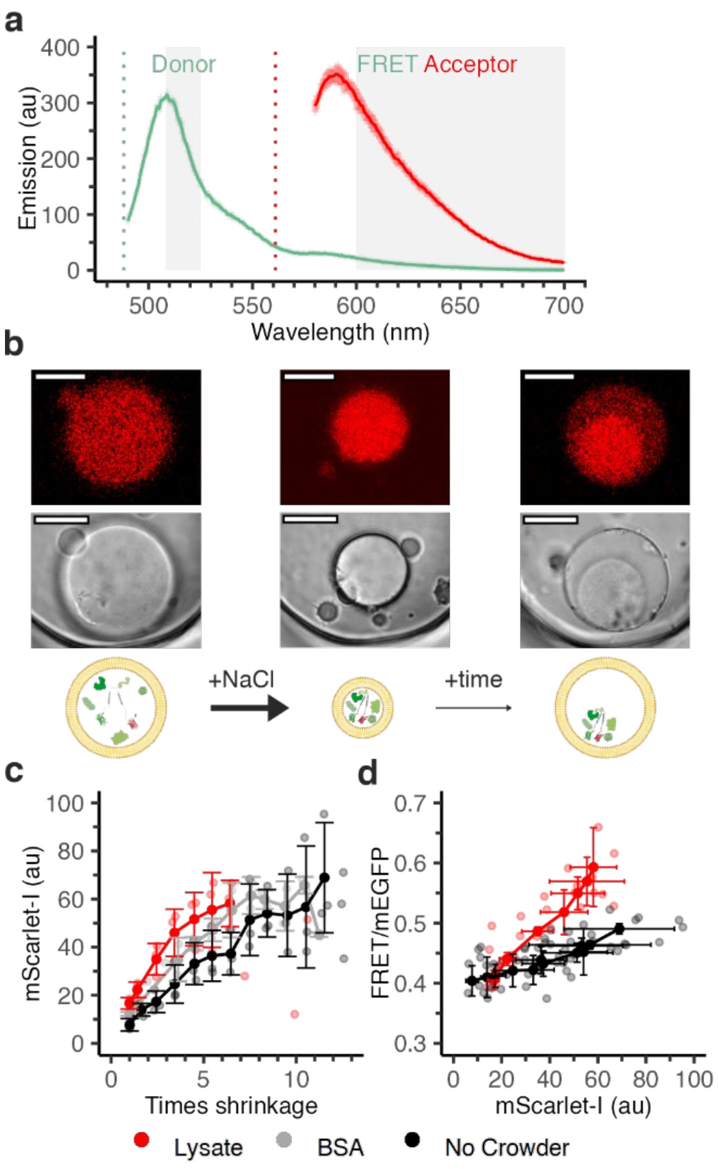

Figure S1: Generation of lysate-crowded GUVs and measurement of crowding. (a) Fluorescent spectra of the crGE2.3 crowding sensor (mEGFP-linker-mScarlet-I) and the donor (ex. 488 nm em. 510 - 525 nm), the FRET (ex. 488 nm, em. 600 - 700 nm) and acceptor (ex. 561 nm em. 600 - 700 nm) channels; (b) Large increase in the external osmolality on lysate-crowded GUVs results in either loss of vesicles (not shown) or lysate heterogeneity inside of the GUVs' lumen. Brightfield and fluorescent (acceptor channel) images of a vesicle before (left) and immediately after (middle) large osmotic upshift. With time the effect of the large non-gradual osmotic upshift can be observed (right). Scale bar 10 µm. (c) The average fluorescent intensity of the acceptor mScarlet-I with volume decrease of GUVs containing lysate (red), BSA (grey), or not crowded GUVs (black), with minimal differences in intensity among conditions. The mScarlet-I fluorescence can be used as a reporter for GUV shrinkage; (d) Plot of the average FRET/mEGFP ratio to mScarlet-I fluorescence for non-crowded (black) and lysate-crowded (red) GUVs. At equivalent mScarlet-I fluorescence levels, the FRET/mEGFP ratio is higher in crowded GUVs than in non-crowded GUVs, suggesting that the increased ratio in lysate crowded GUVs is due to

increased crowding and not to intermolecular FRET. Error bars represent the standard deviation of 3 biological repeats.

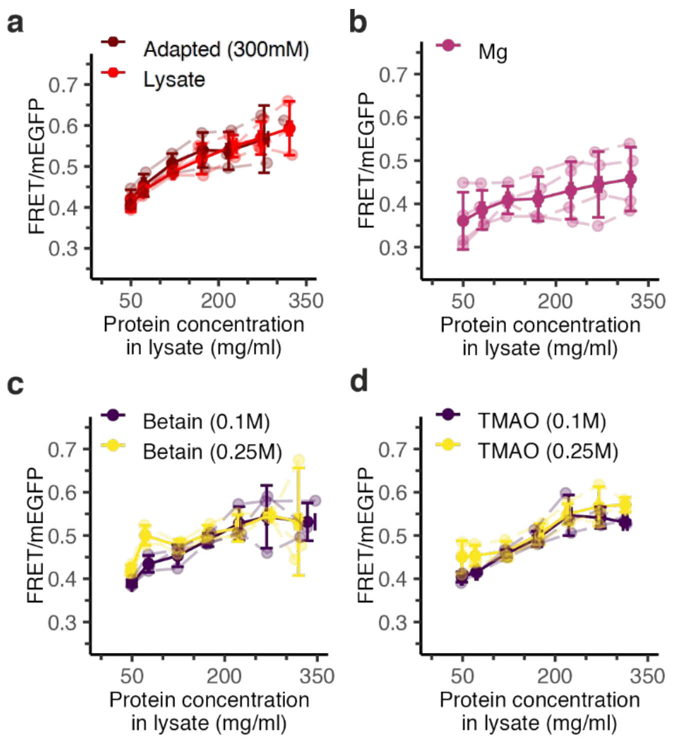

Figure S2: Effect of cosolutes on macromolecular crowding. (a) The FRET/mEGFP ratio of GUVs with lysate from *E. coli* grown in standard LB medium (red) or LB medium supplemented with 300 mM NaCl (brown) showed no difference; (b) Increasing Mg-Glutamate concentration (40 mM instead of 6 mM) lowered the FRET/mEGFP ratio, suggesting that higher Mg(II) levels may influence crowding within the GUVs. Addition of betaine (c) or TMAO (d) at varying concentrations did not produce meaningful changes in the FRET/mEGFP ratio of lysate-crowded GUVs. For (b) and (c), cosolutes were added to the standard lysis buffer (30 mM K-Glutamate, 6 mM Mg-Glutamate, 10 mM NaPi pH 7.4, and cOmplete protease inhibitor cocktail). Error bars represent the standard deviation of 3 biological repeats.

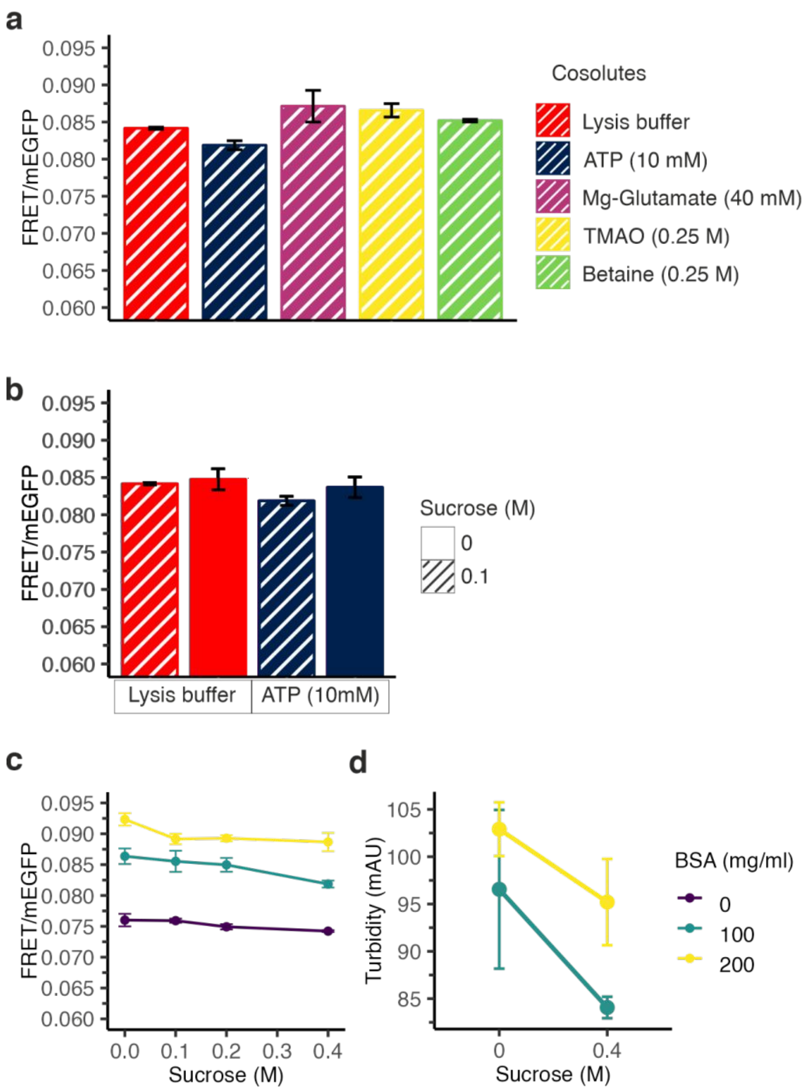

Figure S3: Impact of cosolutes on the crGE2.3 sensor. (a) FRET/mEGFP ratio comparisons for buffers used in crowded GUVs experiments. The addition of ATP to the lysis buffer slightly lowers the ratio, whereas the addition of magnesium glutamate or TMAO slightly increased it; (b) Effect of sucrose on FRET/mEGFP ratio in GUVs lysis buffer with or without addition of ATP, indicating that sucrose does not directly affect sensor readout; (c) FRET/mEGFP ratios in bulk BSA solutions (0, 100, 200 mg/ml) with varying sucrose concentrations (0–0.4 M) show that in the presence of a crowder, sucrose can slightly decrease the ratio; (d) Average turbidity (at 25 °C) of BSA solutions from (c). The samples used in both (c) and (d) contain 10 mM NaPi (pH 7.4), with varying concentrations of BSA and sucrose but without protease inhibitors or other cosolutes. Error bars denote the standard deviation across three biological replicates. Lysis buffer: 30 mM potassium glutamate, 6 mM magnesium glutamate, 10 mM sodium phosphate buffer (NaPi, pH 7.4), and a cOmpleteTM protease inhibitor cocktail.

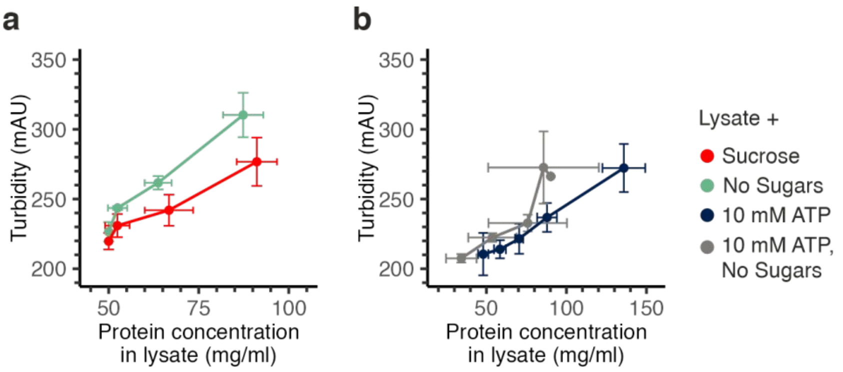

Figure S4: Thermal unfolding and FRAP of concentrated bacterial lysates containing ATP and sucrose. (a) Average turbidity at 25 °C of bacterial cell lysate concentrated in bulk with or without sucrose, shows that the presence of sucrose slightly decreases the turbidity; (b) average turbidity at 25 °C of bacterial cell lysate concentrated in bulk supplemented with ATP, in the presence or absence of sucrose.

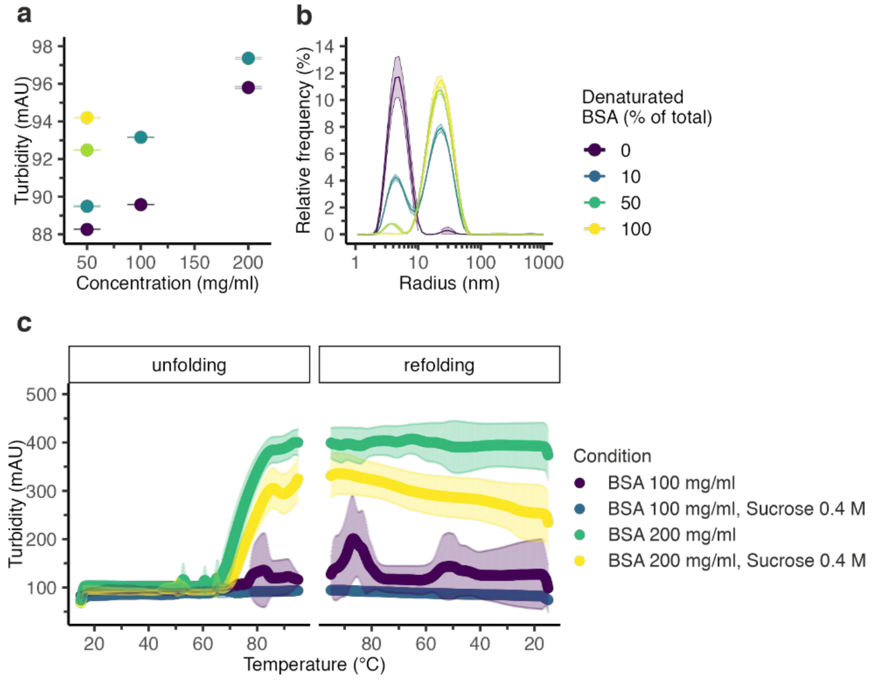

Figure S5: Thermal unfolding and DLS of bulk solutions of BSA. (a) Average turbidity at 25 °C of different concentrations of BSA solutions containing 0%, 10%, 50%, or 100% denatured protein shows that the presence of denatured proteins increases the turbidity; (b) Average size distribution of the BSA solutions in (a). The samples were diluted to 2 mg/ml before the DLS was performed; (c) Average thermal unfolding and refolding profile of BSA solutions containing sucrose (0 M or 0.4 M), shows that the presence of sucrose slightly decreases the turbidity. The BSA solutions were prepared in 10 mM NaPi (pH 7.4) (a and b) and sucrose (c). Error bars represent the standard deviation of 3 biological repeats.

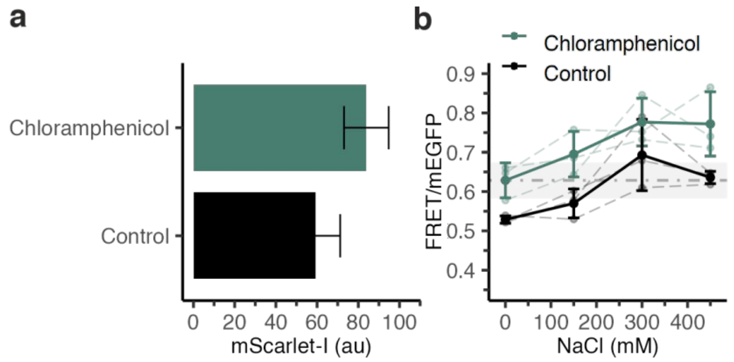

Figure S6: Crowding in *E. coli* cells. (a) Fluorescent intensity of acceptor mScarlet-I in *E. coli* BL21(DE3) cells expressing the crGE2.3 gene. The cells were treated with 0.2 mg/ml chloramphenicol (green) or with an equivalent volume of ethanol (black, final concentration 0.4 % v/v). (b) FRET/mEGFP ratio of osmotically challenged the *E. coli* BL21(DE3) cells in (a). Shading represents the average FRET/mEGFP ratio for unstressed chloramphenicol-treated cells. Error bars represent the standard deviation of 3 biological repeats.

Sequences

DNA Sequence of crGE2.3 crowding sensor for *E. coli*

ATGGGTCATCATCATCACCATCACAAAGGTGAAGAACTGTTTACCGGTGTTGT
TCCGATTCTGGTTGAACTGGATGGTGACGTTAATGGTCACAAATTTTCAGTTA
GCGGTGAAGGCGAAGGTGATGCAACCTATGGTAAACTGACCCTGAAATTTATC
TGTACCACCGGCAAACTGCCGGTTCCGTGGCCGACACTGGTTACCACACTGAC
CTATGGTGTTCAGTGTTTTAGCCGTTATCCTGATCACATGAAACAGCACGATT
TTTTCAAAAGCGCAATGCCGGAAGGTTATGTTCAAGAACGTACCATCTTCTTC
AAAGATGACGGCAACTATAAAACCCGTGCCGAAGTTAAATTTGAAGGTGATAC
CCTGGTGAATCGCATTGAACTGAAAGGCATCGATTTTAAAGAGGATGGTAATA
TCCTGGGCCACAAACTGGAATATAATTATAATAGCCACAACGTGTACATCATG
GCCGACAAACAGAAAAATGGCATCAAAGTGAACTTCAAGATCCGCCATAATAT
TGAAGATGGTTCAGTTCAGCTGGCCGATCATTATCAGCAGAATACCCCGATTG
GTGATGGTCCGGTTCTGCTGCCGGATAATCATTATCTGAGCACCCAGAGCAAA
CTGAGCAAAGATCCGAATGAAAAACGCGATCACATGGTGCTGCTGGAATTTGT
TACCGCAGCAGGTATTACCTTAGGTATGGATGAACTGTATAAAGGATCCGGTG
GTAGCGGTGGTTCAGGTGGTAGTGGCGGTAGTGGTGGCAGCGGTGCAGAAGC
AGCAGCAAAAGAAGCCGCTGCCAAAGAAGCGGCAGCGAAAGAGGCTGCCGCAA
AAGAGGCAGCAGCGAAAGAAGCAGCGGCTAAAGCAGGTTCAGGCGGAAGCGG
AGGCAGTGGTGGATCAGGCGGATCTGGTGGCTCAGGTGCCGAGGCAGCAGCA
AAAGAGGCAGCTGCTAAAGAGGCTGCTGCAAAAGAAGCAGCCGCAAAAGAGGC
AGCGGCAAAAGAAGCCGCAGCAAAAGCAGGTAGTGGTGGAAGTGGCGGTTCC

GGTGGCTCTGGTGGAAGCGGTGGCTCCGGAGTTAGTAAAGGCGAAGCAGTTAT
TAAAGAATTTATGCGCTTCAAAGTGCACATGGAAGGTAGCATGAATGGCCATG
AATTTGAAATCGAAGGTGAAGGTGAGGGTCGTCCGTATGAAGGCACCCAGACC
GCAAAACTGAAAGTTACCAAAGGTGGTCCGCTGCCGTTTAGCTGGGATATTCTG
AGTCCGCAGTTTATGTATGGTAGCCGTGCATTTATCAAACATCCGGCAGATA
TCCCGGATTATTACAAACAGAGCTTTCCCGAAGGTTTTAAATGGGAACGTGTG
ATGAATTTTGAGGATGGTGGTGCAGTTACCGTTACACAGGATACCAGCCTGGA
AGATGGCACCCTGATCTATAAAGTTAAACTGCGTGGCACCAATTTTCCGCCAG
ATGGTCCTGTTATGCAGAAAAAAACCATGGGTTGGGAAGCAAGCACCGAACGT
CTGTATCCTGAAGATGGCGTTCTGAAAGGTGATATCAAAATGGCACTGCGTCT
GAAAGATGGTGGTCGTTATCTGGCAGATTTCAAAACCACCTACAAAGCCAAAAA
ACCGGTTCAGATGCCTGGTGCATATAATGTTGATCGCAAACTGGATATCACC
AGCCATAATGAAGATTATACCGTGGTGGAACAGTATGAACGTAGCGAAGGTCGT
CATAGTACCGGTGGCATGGATGAATTATACAAAGGTGGCACCTAA

DNA Sequence of crGE2.3 crowding sensor for HEK293T

ATGGGCAAGGGCGAAGAACTGTTTACAGGCGTGGTGCCCATCCTGGTGGAACTG
GACGGGGATGTGAACGGCCACAAGTTTAGCGTTAGCGGCGAAGGCGAAGGG
GATGCCACATACGGAAAGCTGACCCTGAAGTTCATCTGCACCACCGGCAAGCT
GCCTGTGCCTTGGCCTACACTGGTCACCACACTGACATACGGCGTGCAGTGCT
TCAGCAGATACCCCGACCATATGAAGCAGCACGACTTCTTCAAGAGCGCCATG
CCTGAGGGCTACGTGCAAGAGCGGACCATCTTCTTTAAGGACGACGGCAACTA
CAAGACCAGGGCCGAAGTGAAGTTCGAGGGCGACACCCTGGTCAACCGGATCG
AGCTGAAGGGCATCGACTTCAAAGAGGACGGCAACATCCTGGGCCACAAACTT
GAGTACAACTACAACAGCCACAACGTGTACATCATGGCCGACAAGCAGAAAAA
CGGCATCAAAGTGAACTTCAAGATCCGGCACAACATCGAGGACGGCTCTGTGC
AGCTGGCCGATCACTACCAGCAGAACACACCCATCGGAGATGGCCCTGTGCTG
CTGCCCGATAACCACTACCTGAGCACCCAGAGCAAGCTGAGCAAGGACCCCAAC
GAGAAGCGGGACCACATGGTGCTGCTGGAATTTGTGACAGCCGCCGGAATCA
CCCTCGGCATGGACGAACTGTACAAAGGCTCTGGCGGCAGCGGAGGTTCTGGT
GGAAGTGGTGGTTCTGGCGGATCTGGCGCTGAAGCCGCTGCTAAAGAAGCCGC
CGCAAAAGAGGCTGCTGCCAAAGAGGCAGCCGCTAAAGAAGCAGCAGCCAAAG
AAGCCGCTGCCAAGGCTGGAAGCGGAGGTAGTGGCGGAAGCGGCGGCTCAGG
TGGTAGCGGAGGATCAGGTGCAGAAGCTGCTGCAAAAGAGGCCGCAGCAAAA
GAAGCCGCAGCTAAAGAGGCAGCTGCTAAAGAGGCTGCTGCAAAAGAAGCTGC
AGCCAAGGCAGGCAGTGGTGGATCAGGCGGCAGTGGCGGCTCCGGTGGATCTGG
TGGAAGCGGAGTTTCTAAGGGCGAAGCCGTGATCAAAGAATTCATGCGGTT
CAAGGTGCACATGGAAGGCAGCATGAATGGCCACGAGTTCGAGATTGAAGGCG
AAGGCGAGGGCAGACCTTACGAGGGAACACAGACCGCCAAGCTGAAAGTGACC
AAAGGCGGCCCTCTGCCTTTCAGCTGGGACATTCTGAGCCCTCAGTTTATGTA
CGGCAGCCGGGCCTTCATCAAGCACCCTGCCGATATTCCCGACTACTACAAAC

AGAGCTTCCCCGAGGGCTTCAAGTGGGAGAGAGTGATGAACTTTGAGGACGGC
GGAGCCGTGACCGTGACACAGGATACAAGCCTGGAAGATGGCACCCTGATCTA
CAAAGTGAAGCTGCGGGGCACCAACTTTCCACCTGATGGCCCCGTGATGCAGA
AAAAGACCATGGGCTGGGAAGCCAGCACCGAGAGACTGTATCCTGAGGATGGC
GTCCTGAAGGGCGACATCAAAATGGCCCTGCGGCTGAAGGATGGCGGCAGATA
CCTGGCCGATTTCAAGACCACCTACAAGGCCAAGAAACCCGTGCAGATGCCTG
GCGCCTACAACGTGGACAGAAAGCTGGACATCACCAGCCACAATGAGGACTAC
ACCGTGGTGGAACAGTACGAGCGGAGCGAAGGCAGACACTCTACAGGCGGAAT
GGATGAGCTTTACAAAGGCGGGACCTAA
